# A Qualitative Analysis of Emotional Facilitators in Exercise

**DOI:** 10.3389/fpsyg.2016.01296

**Published:** 2016-08-29

**Authors:** Benjamin Wienke, Darko Jekauc

**Affiliations:** ^1^Department of Sport and Exercise Psychology, Humboldt University of Berlin, BerlinGermany; ^2^Department for Sport Science, University of Konstanz, KonstanzGermany

**Keywords:** emotions, grounded theory, exercise maintenance, perceived competence, belongingness, novelty experience, exercise intensity

## Abstract

Although previous research has shown that emotions are consistently associated with sport and exercise behavior, the working mechanisms are not understood to the extent of creating an intervention. The aim of this study is to identify situations and aspects of recreational sport and exercise, which lead to positive emotional reactions in people taking part in regular and long-term exercise. In this study, 24 adults (12 female, 12 male) distributed over three age groups (young, middle, and late adulthood), took part in recreational sports (individual or team sport) for at least 5 years. Semi-structured in depth interviews with questions about sport and exercise habits, long term participation and emotional response in a sporting environment were conducted in order to ascertain those situations and aspects of the exercise program triggering positive emotions. Interviews were transcribed verbatim and followed Grounded Theory principles. Emerging concepts were grouped and merged into different categories representing the key aspects of sport and exercise. Four factors were identified which are associated with the emergence of positive emotions in recreational sport and exercise. Firstly, perceived competence is one of the major factors influencing emotions during exercise and can represent individual and collective success and progress, competition and challenge. Secondly, perceived social interaction is another factor comprising of all sorts of peer-related aspects such as communication with others, being part of a group and creating close relationships or friendships. Thirdly, novelty experience in contrast to other none-sporting activities such as work, family or other leisure activities was another factor. The last factor found was the perceived physical exertion comprising of the degree of exhaustion, a possibly delayed turnaround in the emotional response and the aspect of sport being a physical compensation for everyday sedentary life. The results of this study provide the starting point for the development of interventions to enhance positive emotions in sports in order to increase maintenance and adherence to recreational sport and exercise.

## Introduction

Evidence shows a wide range of health benefits from exercise and physical activity (Paffenbarger et al., 1993; Mensink, 2003; Rütten et al., 2005; Krug et al., 2013; Reiner et al., 2013), but also poor compliance and adherence to exercise programs (Mensink, 2003; Ekkekakis and Lind, 2006; World Health Organization [WHO], 2010; Krug et al., 2013; Eurobarometer, 2014). These health improvements only persist due to regular participation in physical activity and exercise whereas a significant decline or drop out causes a decrease or complete loss of initially gained benefits (Mujika and Padilla, 2000a,b). Regardless of people knowing this necessity to continue exercising, the majority of the population remains physically inactive even those who are younger (Jekauc et al., 2012). Furthermore, empirical evidence has identified high dropout rates of up to 50% in exercise programs, within the first 6 months (Dishman and Buckworth, 1996; Annesi, 2003) and a common relapse to lower or no levels of activity after the exercise program has finished (Amireault et al., 2013). Effort has been made to encourage behavior change (Biddle et al., 2012) with marginal success (Rhodes and Pfaeﬄi, 2010). Even though insights about the motivational process of initiation of physical activity and exercise is advanced, there is a paucity of research which investigates the maintenance of such activities (McAuley et al., 2007; van Stralen et al., 2009; Kwasnicka et al., 2016) as well as a lack of effective practice (Amireault et al., 2013). Previous research indicates that the initiation of physical activity and exercise has different influences (e.g., strategic planning and recovery self-efficacy; Schwarzer et al., 2007) in comparison to the maintenance (habit, environmental, and social influences; Kwasnicka et al., 2016) of it (see also Biddle and Mutrie, 2008). Therefore there is a high interest in identifying key factors which affect physical activity and exercise maintenance such as specific facilitators (Amireault et al., 2013). Although recent findings support the notion of facilitators which foster behavior maintenance (previous physical activity habits, positive attitude, expectations; Amireault et al., 2013; perception of competence, social affiliation, environmental conditions, sense of autonomy, specific physical activity; Rhodes and Kates, 2015), the capability of established theoretical frameworks is fairly limited (Rhodes and Pfaeﬄi, 2010; Amireault et al., 2013). Indeed, most factors are based on cognition, but established cognitive theories and models can only explain parts of people’s behavior (Jekauc et al., 2015; Kwasnicka et al., 2016) revealing a lack of theoretical foundation. Perhaps diverting attention to other domains such as facilitators of emotions in the context of sport and exercise is more promising (Williams, 2008; Rhodes and Kates, 2015) and may lead to a better understanding, which despite years of research is still limited (Rhodes et al., 2009).

To succeed using this approach a clear definition is crucial to distinguish between different terms and constructs used (Tuson and Sinyor, 1993). According to Caspersen et al. (1985) *“physical activity is defined as any bodily movement produced by skeletal muscles that results in energy expenditure” […]* which *“can be categorized into occupational, sports, conditioning, household, or other activities”* (p. 126). Instead, sport and exercise are subcategories of physical activity, which are often planned, structured, and repetitive with the purpose of improving or maintaining physical capability (Caspersen et al., 1985). Since sport and exercise are subsets of physical activity, evidence drawn from sport or exercise might be valid for each other and broadly for physical activity but not the other way round. Following the approach from Buckworth et al. (2013), this article is mostly focused on exercise because the majority of researchers used methods that fit the definition of exercise mentioned above, to measure physical activity. Since classic competitive sports are expected to have a specific emotional pattern (e.g., influenced by a higher goal orientation or different expectations such as external rewards), a look at recreational sports might result in a broader insight.

The same procedure is required for the differentiation of affects and emotions. According to Russell and Feldman Barrett (2009) the *core affect* (because it is considered to be basic or irreducible; Ekkekakis, 2003) is characterized as a “*neurophysiological state consciously accessible as a simple primitive non-reflective feeling most evident in […] emotion but always available to consciousness*” (p. 104) such as pleasure and displeasure, tension and relaxation, or energy and tiredness (Ekkekakis, 2012). Core affect can occur on its own or as a part of emotion, e.g., like pride which is feeling good about oneself whereas ‘*feeling good’* represents the core affect and ‘*about oneself’* the other, cognitive, component (Russell, 2003). Thus emotions are a kind of affective state, but they do not apply vice versa. Making reference to Russell and Barrett (1999) and Ekkekakis (2012) simplifies the definition of emotions that are elicited by and are a reaction to and about a certain stimulus, with its cognitive appraisal involved as an elemental characteristic and reveals a more complex phenomenon in comparison to basic affect (Ekkekakis and Petruzzello, 2000). The previously mentioned example of ‘pride’ represents an emotion which includes all these criteria mentioned before and demonstrates the close relation between the two concepts of affect and emotion. In this article, the focus is primarily on emotions and not affects not only due to the qualitative design of the study.

The aim of the present study is to identify facilitators which affect the emotional response to recreational sport and exercise and thus the maintenance of both. Research about the role of emotions in relation to sport and exercise or facilitators is sparse (Rhodes and Kates, 2015) and the few models that exist focus on leisure time activities that seek pleasure, enjoyment, and fun (Rhodes et al., 2009). Positive affects are one of the fundamental basics in order to understand the nature of enjoyment (Scanlan and Simons, 1992) which in turn reinforce behavior (Rhodes and Kates, 2015; Kwasnicka et al., 2016) and enhance participation and maintenance in sport and exercise (Stenseng et al., 2015). Thus facilitators for positive emotion during exercise must be identified to prompt the development of interventions charged with stimuli which affect one or more facilitators to ensure that each individual experiences positive emotion (for details see the results section below; Scanlan and Simons, 1992).

## Materials and Methods

A qualitative study was conducted to identify facilitators that affect the emotional response to recreational sport and exercise.

### Participants

In order to ensure heterogeneity of the sample the participants were evenly recruited across three factors: age, sex, and type of exercise. Age was divided in three age categories: 18–34 years (AG1), 35–59 years (AG2) and older than 60 years (AG3). Sex (male vs. female) and type of exercise (group vs. individual) consisted of two categories. Combining these three factors (3 × 2 × 2) 12 cells emerged. For each cell, two participants were recruited. Thus, the sample consisted of 24 participants. All participants took voluntarily part in this study and were recruited in Berlin as native German speakers. Ethical approval for the study was provided by the Humboldt University and all participants signed an informed consent form at the beginning of the study.

Participants were involved in formal recreational sports such as soccer, volleyball, or swimming and informal recreational sports like climbing, martial arts, or paddling; organized in sport clubs, training groups, or exercising on their own. None of the participants were a high-level-competitive or professional athlete or worked in a job that involved sport. All participants described their actual level of engagement in popular sports as more or less ambitious. The average training frequency was three times a week (mean: 2,98; *SD* = 1,84) and the duration was around 90 min (mean: 94,04; *SD* = 34,42). Compared to their age, participants reported an involvement in recreational sports of around 70% of their lifespan (e.g., age of 28 and 22 years of sport participation results in 78, 57% of engagement; mean: 69,89; *SD* = 22,77) with a minimum of 6 years engagement. Of the 24 participants, there were even 12 that took part in competitions (e.g., league game or small tournament), five participated in competitions previously and seven had never participated in any competitions.

### Data Collection

Students of Sport Science of the Humboldt University of Berlin through acquaintances or friends recruited suitable participants without any deeper relationship to the interviewer. At the start of each interview, relevant information about the study, the procedure and the data usage were given to the participants which had agreed to be interviewed and recorded. Data collection was made via voluntary, non-directive, face to face interviews, which where standardized using a semi-structure interview manual according to Tong et al. (2007) which refers to a gradual three level approach (three types of questions from the general to the specific) in a quiet environment. Participants were asked open-ended-questions about their experiences, habits, and emotions in the context of sport and exercise. Firstly, questions remained general in order to be able to get to know the participant, e.g., “*How did you get into sport and exercise?*” “*What is your first memory in the context of sport and exercise?*” “*Which of these kind of sports/or what kind of exercise do you do regularly?”* The second step was to ask about their habits and how they emerged. *“What significance does regular exercise have in your everyday life?” “How much do you have to think about deciding whether to exercise?” “If you decide not to exercise, could you describe the inner monolog with yourself any further?” “Would you miss something after quitting your sport/exercise?”* The final part of the interview contained questions especially about emotions within exercise and sport: *“What kind of feelings do you experience whilst you exercise?” ”In which kind of situation do you experience the most enjoyment?” “How significant is the experience of pleasure to you, to be able to keep on exercising?”*

In addition to the participant and the interviewer, a third person attended to write minutes of the interview, which lasted approximately 20–40 min. Referring to GAT (“Conversation Analytic Transcription System”; Selting et al., 2009) the interviews where transcribed verbatim.

### Data Analysis

**FIGURE 1:**
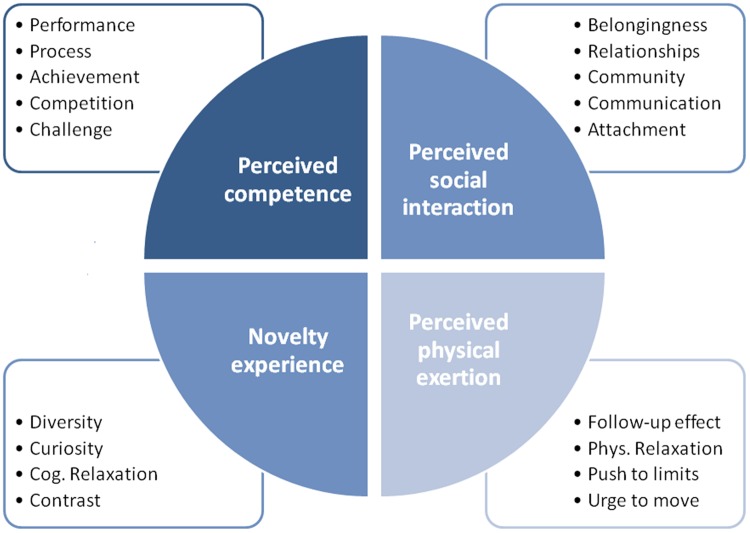
**Concepts which form each of the four categories**.

Verbatim transcripts of the audio recordings were used in content analysis related to the Grounded Theory (Strauss and Corbin, 1996). The first step was the open encoding of the full text of the whole 24 interviews. Therefore the interviews were read completely by three different independent researchers and thoughts were written down in memorandum. Relevant information was grouped into different concepts. This working phase was done independently by two researchers, who met for a weekly meeting to compare and to discuss their findings and stayed as close as possible to the script in order to avoid bias. All concepts were grouped within categories (e.g., performance, process, achievement, competition, and challenge in the context of positive emotions were summarized in the category*perceived competence* (*PC*; **Figure 1**) and were checked for connections between each other in the axial encoding process. In that second step categories that seemed synonymous were merged into one so that only the varying categories remained [e.g., categories such as *closeness to nature* and *curiosity and otherness* were merged into *novelty experience* (*NE*)]. The last working step was selective coding to ensure the integrity of the four categories by searching for appropriate concepts and by analyzing the context in which they were mentioned. The final data was organized accordingly into categories and subcategories in order to be able to formulate the theory that is the focus of this study.

## Results

**FIGURE 2:**
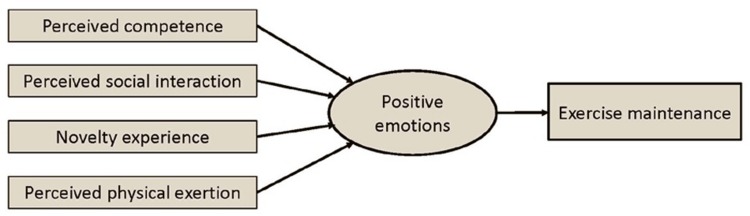
**Facilitators of positive emotions in a sporting environment**.

The majority of participants (22 out of 24) with their long lasting engagement in recreational sports and exercise referred to positive emotions related to this activity. For a better understanding, it is crucial to discover facilitators for these positive emotions to increase sport and exercise involvement and its maintenance. Four different factors with a high to moderate impact on the emotional experience could be revealed (**Figure 2**). The strongest and the most frequently mentioned concepts refer to the category *PC* which was mentioned in 22 out of 24 interviews. The second conceptual group was related to *perceived social interaction (PSI)*, which was mentioned in 19 out of 24 interviews. The third group was related to NE and was mentioned in 18 out of 24 interviews. Lastly, the physical dimension *perceived physical exertion (PPE)* was found in 14 out of 24 interviews. Whereas three of these facilitators are psychological, one is also affected by interoceptive signals. According to the three independent factors (sex, age, and sport type) some differences have occurred as discussed below (**Table 1**).

**Table 1:** Occurrence of concepts related to positive emotions corresponding to one of the four categories.

Groups (sample size)	Concepts (24)	Male (12)	Female (12)	Individuals (12)	Team sports (12)	AG1 18–34y (8)	AG2 35–59y (8)	AG3 +60y (8)
Perceived competence	22	11	11	11	11	8	7	7
Perceived social interaction	19	9	10	9	10	7	6	6
Novelty experience	18	8	10	9	9	5	6	7
Perceived physical exertion	14	10	4	6	8	7	5	2

### Perceived Competence

Firstly, PC was found to be the most influential factor in the emotional experience in recreational sport and exercise, -feeling competent and capable was often combined with positive emotions. PC represents the own individual sense of how well one is performing in a particular situation in relation to exercise, in the present, retrospectively or in the future. Whilst performing well during a task, being successful or winning a game, mastering a challenge, achieving a goal or making progress created positive emotions in all participants such as pleasure and enjoyment; there was also often excitement or pride:

“When I think back to how we won the championship I remember being happy for weeks”; “It is simply awesome when you are allowed to shoot the free kick and are able to fool the wall–I always really look forward to doing that”; “If something really cool worked out, then you are very happy about it. Sometimes you are also proud when you are making progress. After our tournament I was proud of my good results.”

Perceived competence is not only binding on the individual performance; collective accomplishments were also perceived and evaluated:

“If you manage to score or prevent a good move being successful or if you see a good move which you saw 30 years ago done by somebody and he replicates that and you comment “hey you didn’t forget that.” These sorts of things makes you happy or happy for the others”; “I am also happy for my teammates when they are doing well and sometimes even when the opponents do something great.”

Only two participants did not refer to any concepts concerning PC as a facilitator for enjoyment and pleasure in recreational sport and exercise. Furthermore there were no cues among the interviews for a negative emotional response to PC or a positive response to a lack of PC. Also sex did not have an impact on PC among the interviews. Men and women mentioned PC in the context of positive emotions equally (both 11 out of 12 interviews). The same result was shown in the individual and team-sport participants (both 11 out of 12). Only the age factor showed a very small variation. In AG1 everybody mentioned concepts related to PC. In both other groups (AG2 and AG3) only seven out of eight mentioned PC as a factor related to pleasure and enjoyment.

### Perceived Social Interaction

Secondly, PSI is another important factor that influences emotional experience in a sport and exercise-related environment and was mentioned in 19 out of 24 interviews. Many participants appreciate the company of others in the context of recreational sport or during exercise. To have social bonds with others or exercise with them triggers positive emotions, whereas being on your own does not (in category 3, NE, being alone triggered positive emotions due to cognitive rest or relaxation). The PSI category comprises of all sorts of peer-related aspects like communication with others, to be part of a group and to create close and warm relationships with others such as companionship or friendship. Meeting friends and teammates, spending time together or exercising together, communicating, and socializing made participants experience positive emotions. In other words, accompanied participants described themselves and especially their emotional state more positively and enthusiastically than in situations where they exercised alone without friends or peers:

“Because it is so incredibly fun and (despite finding that every now and then I think about quitting) I realize that it is so much fun and such a pleasurable experience and I experience amazing things with my teammates and that’s the thing that keeps me going–not so much the success. It is the community, great experiences, and good feelings.” “Swimming was enjoyable, but if you are part of a team, it is a lot more fun” ”Climbing in a group is like basketball–I really look forward to these amazing days, its similar to snowboarding with several friends a few days–I really enjoy that“ “The fun and pleasure is enormous and we all stayed together because we functioned very well as a group” “What one wants to have regularly is to meet the people who are also there and with whom you have fun with and love to train with and everything that goes with it.”

Perceived social interaction as a facilitator of enjoyment and pleasure has reasonable homogeneity among the cells sex, age, or sport-type. Almost the same frequency was found in both sport-types (individual or team): 9 out of 12 individuals compared to 10 out of 12 team sports interviews mentioned PSI related concepts. However, one difference must be considered: the dissimilar description pattern of PSI from the different types of recreational sport. Both, individual and team-sport groups described PSI in a sports-related environment, e.g., meeting people before or after exercise as well as for other non-sporting purposes:

“*The aim is to have fun with the other girls, and they organize a lot of things to do outside of handball. For them, it is like a replacement for family.” “Well I have to say that the camaraderie and the team spirit is quite strong. You travel together to tournaments or drink a beer or go to eat together after training. Some of them also came to my birthday. We also sometimes met at our trainers place to watch a game on his television. That’s pretty cool.”*

However, participants from team sports also described PSI during exercise or at least directly related to it:

“A smile on your face and cheering on your teammates, having fun together and a good time”; “It’s really the team spirit which entices me, this kind of sport is fascinating to me.”

On account of this, PSI triggers positive emotions directly through the involvement with others such as teammates or indirectly through the company of others. However, it was the environmental concepts that were the most commonly described. In five interviews participants did not mention any concepts concerning PSI as a facilitator for enjoyment and pleasure in sport and exercise. However, the majority of the participants described positive emotions in situations, when they were in the company of significant others. None of the participants stated negative emotions whilst they were exercising with others (the only exception was at the start of a marathon among hundreds of other people which hindered the performance). The difference between male and female participants was very weak (male 9 out of 12 and female 10 out of 12 participants); this also applied to the three age groups (AG1: seven out of eight, AG2 and AG3: six out of eight). Only two elderly participants explicitly mentioned being fine to exercise on their own, but didn’t exclude company *per se*.

“*Well I also like to go (exercise) alone and enjoy the time I have for myself.”*

This is a reference to the third category that describes relief and relaxation when people are able to ‘switch off their heads’ and let their thoughts go.

### Novelty Experience

Thirdly, NE in contrast to the daily life routine was another factor influencing some of the emotional experiences in recreational sport and exercise mentioned in 18 out of 24 interviews. Whilst typically most kinds of exercise are dissimilar to most work-, family-, or other none-sporting commitments and activities, the divergent character of NE lies in the nature of sport and exercise itself. The difference is not limited to activities *per se* and can be found in many dimensions, e.g., the diversity of surroundings, the character of certain people involved in sports or the alternating demand of exercise–sometimes playful or challenging. Concepts such as enjoying the landscape and countryside, to be outside and do outdoor activities- in contrast to most indoor activities, to relax on a cognitive level, to gain new experiences, or to do something non-productive just for fun; curiosity or variety were all grouped into NE.

“And when it was lightly raining and you were completely alone out on the water and you are paddling slowly and there is hardly any noise… you really hear the rain on the water, like singing. It’s wonderful–at least that’s what I think.” “On the one hand I find the snow and the mountains amazing, as well as the landscape which is thrilling and the sun–which is enormous,” “the otherness of the game compared to daily life, most emotions are triggered by that.” “It is when you are on high up in the mountains on a slope and you get this feeling of free movement whilst descending, the swinging to the left and right, and then moving to adapt to the course of the landscape. It is then I get the feeling of pleasure and satisfaction.”

Another part of NE is the cognitive relaxation component. To switch off the brain and let thoughts go, triggers positive emotions in and concentrates the mind on something other than stress and is a contrast to the focus on daily life. Amongst the interviews exercise was often mentioned as an important tool to compensate other influences. NE is only one element of this method, representing the cognitive aspect and must be distinguished from PPE (fourth category), which is related to a physical dimension:

[When exercising] *“Then I’m not able to think about my job anymore. When I was on the water at the weekend and returned to study on Monday, my head was totally free. I only realized that much later. The mental concentration required while you are sailing- you have to focus on the route, the rules, the environment, and adapting to the elements–had a tremendous recuperative effect on me and I was filled with inner satisfaction and peace.” “That moment of diving into the water I find really off-putting but afterward it is pure relaxation. Nothing to think about, nothing else to do. Because of that, I often miscount. Only when I leave the water and have to dry myself, does it turn back to being bad”; “it’s more like turning off from your job. That’s my goal, switching off from the office job and daily routine and to have a bit of fun doing something you want to do.” “You can turn off your head on the track and run because you don’t have to do anything in particular and you can simply let your thoughts go.”*

In comparison to the male participants (8 out of 12), female participants referred slightly more to concepts related to NE (10 out of 12). However, there were no differences between the individuals and team-sport groups (individuals- 9 out of 12; team sport- 9 out of 12). Among the three age groups a slight increase in the amount of statements about the concepts was found (AG1: five out of eight; AG2: six out of eight; AG3: seven out of eight).

### Perceived Physical Exertion

The last factor found was PPE. Concepts related to this category were found in 14 out of 24 interviews. In addition to its psychological component, this category exclusively contains a physical dimension of the body, while the other three are on a pure psychological level. PPE is the phenomenon that sport and exercise *per se* but also fatigue and exhaustion caused by such activities generates a specific and desired emotional response. This effect was described whilst exercising and especially when pushing oneself to the limit but was mentioned more after exercising.

Similarly to NE, this category is also a contrast to the daily life routine with its typical lack of movement and physical demand. Therefore PPE represents an inner drive for movement, the pleasure after exercise or the physical balance desired after long periods of sitting or leading sedentary lifestyle *per se*. Participants referred to positive emotions like pleasure and enjoyment in situations where they could satisfy this drive for movement or do other things that were different to the daily routine such as sport. Even the perception of exhaustion due to strenuous exercise, the opportunity to burn off energy or the growing muscle soreness triggered positive emotions:

“The feeling of pleasure during sport is when you are exercising and push yourself, you get feelings of happiness. For me, when I know that I can work out and when I am finished, I pushed myself and afterward I am actually more satisfied and happy.” “Well running sometimes feels a bit like torture but nevertheless I feel happy during the run and afterward too.” “And if something went wrong you are able to really push yourself to the limit when doing sport and mostly you always feel better afterward. Because of that it is a really good way to balance out my job.”

Another component of PPE is the inner need for movement. People described an impulse to exercise but not necessarily for a certain purpose or a specific goal. They responded with positive emotions whilst fulfilling the desire to exercise:

“It has an enormous impact and if I’m not exercising one day I notice that I don’t feel as well as I normally do. I need that very much at least once a day, if not more often.” “When I arrive at home I feel well balanced, if I wouldn’t exercise I would sit at home with the same incomplete work and be frustrated and nervous and in a bad mood. I would then start to concentrate more on the smaller things that aren’t so important.” “Well the regularity is very important for me, I’m angry if my training is canceled and then I realize that I miss it because I’m not balanced.” “I’m used to it. If I was completely without exercise …well there are some periods in between where you are forced to interrupt exercise but then I miss it quite a lot.”

To underline this point a common theme about missing exercise was very strong among the interviewees. All participants mentioned, that they would miss their familiar sports (24 out of 24).

One crucial part of PPE is the follow-up or rebound effect. In these interviews all participants stated positive emotions after exercising (but only if those emotions were related to a physical response or effect such as exhaustion and not due to evaluation, for example due to a lost match):

*“When I*’*m not exercising, I recognize after a while that I’m not feeling physically well. I certainly need to push myself physically and afterward the endorphins are released. You always feel better after having exercised.” “I am simply more relaxed and less tense when I’m physically exhausted and you have a total different feeling about life. You sleep very well because you have pushed yourself physically and because it cleared your head from thinking about your job you have a better feeling about life. ” “Well emotionally balanced and after exercise a happy exhaustion. Therefore, satisfied, happy, and physically exhausted.” “It is like a happy exhaustion after a training session. Frequently, I recognized then that I am finished with the training session due to these feelings. It’s a kind of very physical happiness.” “When you are finished you think about what you have done and that you managed to overcome your doubts and do it, which is probably like a reward system in itself. Well after exercising you have, 99% of the time, positive feelings afterward.”*

There was a strong difference between male and female participants. 10 out of 12 men reported situations or concepts related to PPE while only 4 out of 12 women did. The need to move, push oneself to their limits, be physically exhausted, or to get the positive emotions afterward was substantially stronger among male participants. The factor sport type, only slightly differed. Individual-participants mentioned 6 out of 12 times concepts related to PPE whilst in 8 out of 12 interviews team participants mentioned it. According to the age factor a strong decline occurred from AG1 (seven out of eight) via AG2 (five out of eight) to AG3 (two out of eight).

## Discussion

To change sport and exercise experiences and thus behavior, it is crucial to identify the major influential factors. As described above there is no doubt that positive emotions are major reasons to adopt and maintain corresponding activities (Jekauc, 2015; Rhodes and Kates, 2015; Stenseng et al., 2015), while a lack of positive emotions leads to dropping out (Scanlan and Simons, 1992). Furthermore perceived enjoyment is a key factor for commitment to sport (Scanlan and Lewthwaite, 1986; Scanlan et al., 1993) and reflects a positive emotional response like general feelings (e.g., pleasure) to the sporting experience (Scanlan and Simons, 1992). This study also provides strong evidence for the assumption, that positive emotions such as fun and enjoyment encourage maintenance of recreational sport and exercise in the long term. Participants even referred to positive emotions in a sporting context occasionally without being directly asked (aside from the specific questions). To enhance participation in recreational sport and exercise, commitment and adherence, a better understanding of factors which makes this experience more enjoyable is crucial, starting with the general to the specific (Scanlan and Simons, 1992; Ekkekakis and Petruzzello, 1999).

According to Weiss and Raedeke (2004) the factors *PC, enjoyment*, and the *social environment* impact adherence and maintenance of physical activity among young people. Similarities were described by Scanlan and Simons (1992) with *PC and challenge, social interactions, elements of activity itself*, and *extrinsic rewards* as mediators which often arise in research. In this article, categories similar to the factors *competence, elements of activity and social environment* and, respectively, *social interactions* were found. *Challenge* is included in the first category: PC. Concepts concerning *extrinsic rewards* were not found during the interviews. Similarly to the factors mentioned above, the four categories found in these interviews with an impact on emotional experience are *PC*, *PSI*, *NE*, and *PPE*. Concepts referring to these facilitators occurred frequently among all twenty four interviews suggesting also a dynamic interplay.

### Perceived Competence

Perceived competence is one category which is deeply anchored in the character of sport and exercise not only because of the Olympic motto, “Citius–Altius–Fortius” but because in despite of progression, many appropriate activities require special skills to perform them. Positive emotions like fun enhance habits or channel positive behavior change (Rhodes and Kates, 2015; Kwasnicka et al., 2016) while negative emotions strengthen actions to avoid something. PC therefore is a facilitator to evolve toward a certain skill level (Deci and Ryan, 2000; Ekkekakis et al., 2005; Cohn and Fredrickson, 2006) as well as helping to sustain this ability as a result of the fun and enjoyment experienced during sport and exercise. The importance of PC as a facilitator for positive emotions is underlined due to it commonness and robustness among all interviews regardless of the independent factors. Furthermore it might reinforce itself since the perception of competence result in positive emotions which in turn support optimal performance (if perceived as convenient; McCarthy, 2011).

White (1959) was the first, who described the interaction between competence and enjoyment. He proposed an intrinsic drive of an individual to deal effectively with the surrounding environment and the positive emotions after doing so (Harter, 1978). In his opinion *effectance motivation* just aims for the feeling of efficacy, not for any consequences (White, 1959). As described in his basic model, this type of motivation leads to mastery attempts in a certain domain. After a successful attempt and PC, a feeling of efficacy arises, accompanied by pleasure, which in turn increases or at least maintains *effectance motivation* (White, 1959; Harter, 1978).

Based on White’s (1959) concept of *effectance motivation*, Harter (1978) developed the competence motivation theory which describes the intrinsic striving of a child to develop competence in certain domains and to achieve mastery level. Related to the amount of competence motivation the child will search for correspondence between challenge and expected success. If the person succeeds in those mastery attempts with an optimal amount of challenge, they will experience PC and control as well as a positive affect response. Significant others may also enhance these perceptions giving social support in different ways and indirectly influence the emotional response. In sum these processes increase the desire “*to continue being effective*” (Weiss and Raedeke, 2004; S.227). It is reasonable to assume that this process applies for the most part equally in older individuals–with its usual characteristic structure but with different intensities of influential behavior and habits.

In summary, in a recreational sport or exercise -related environment, PC is undoubtedly a major facilitator on emotions with a robust effect on the emotional response and furthermore essential for any type of motivation (Deci and Ryan, 2000). Connections to PSI, NE and PPE were found (e.g., collective success, flow, and push to the limit).

### Perceived Social Interaction

Humans do have a natural need to establish and sustain close and warm relationships (Maslow, 1968). Forming these bonds as well as amplifying them is ensued by positive emotions such as pleasure and happiness. To trigger these emotions people need frequent social interactions with others, ideally experienced as pleasant and enjoyable. However, more importantly is the perception of belonging and the social tie itself rather than mere social contact (Baumeister and Leary, 1995). Furthermore, a consistent and long-term interaction with familiar people is experienced as more satisfactory than changing partners or strangers. This effect increases due to mutuality, which is supportive but not necessarily essential (Clark, 1984; Baumeister and Leary, 1995). The facilitator PSI may promote positive emotions during sport and exercise and afterward. Most kind of sports provide an appropriate environment for social interactions because many people tend to exercise with others. According to Coon (1946), affiliating in groups is inherently instinctive rather than practical (e.g., cost-effective, same time, interests, etc.). In addition, it seems that behavior change from individuals within a group is more promising than changing them alone (Lewin, 1951; Kwasnicka et al., 2016). Perhaps positive motivation from others who enjoy the physical activity might enhance the attitude toward it and the effect is cumulative. Although this collective reinforcement might also apply to negative emotions, it is reasonable, that the majority of emotions whilst exercising are positive. As the participants stated, –‘otherwise people won’t continue with an unpleasant activity.’ However, PSI as mentioned by the majority of the participants enhances their emotional experience in the context of sport and exercise. This indicates that the need for attachment and belonging is affected which can be considered to be fundamental (Baumeister and Leary, 1995; Lavigne et al., 2011). People experience pleasure and enjoyment when creating new relationships such as entering a new sport, team, or club, but this was even more so when reinforcing or sustaining existing ties (Baumeister and Leary, 1995). On the other hand, it seems that positive emotions also influence belongingness (Stenseng et al., 2015) and due to their reciprocal relationship are self-energizing. Many participants reported, that either their parents or their friends were the initial cause of starting their engagement in sports and exercise–both figures in which the participants have a bond and attachment. PSI might convert recreational sport and exercise into something more attractive. Participants also underlined positive emotions while exercising together with friends or being accompanied by them in an exercise context. These emotions might arise due to the exercise itself, due to the satisfaction of the need to belong or due to a combination of both. Although participants thought that their sporting-activities were enjoyable, they explained that the intensity of positive emotions was weaker when exercising alone. Similarly lower levels of enjoyment were reported from participants who spent time together without recreational sport and exercise. The highest degree of positive emotions occurred when exercising along with others or directly together. Recent findings suggest, that a basic function of emotion is to encourage the formation and sustenance of relationships (Baumeister and Leary, 1995). Because people are happy and also resist the dissolution of relationships, they participate more often and are more likely to continue with recreational sport and exercise much longer than they normally would without these social ties. Along with recent evidence this study confirms the connection between belongingness and positive emotions in recreational sport and exercise (Stenseng et al., 2015). Furthermore relationships with NE and PPE were found (interaction with teammates, reduced negativity of high intensity).

### Novelty Experience

According to the Latin proverb, ‘variatio delectat,’ variation can be seen as a source of positive emotions. Furthermore, the distraction hypothesis may provide an explanation for the ‘feel better’ effect normally associated with exercise. It assumes, that distraction from stressful stimuli rather than the physical exercise itself enhances positive emotions (Morgan, 1985). Participants often reported, that they enjoyed situations in which they could ‘turn off their head’ and let their thoughts go. However, research could not verify a stronger impact of exercise in terms of distraction in comparison to equivalent activities such as rest or relaxation on emotional states (Yeung, 1996). It seems that the distraction hypothesis is not able to explain why participants choose exercise rather than rest or relaxation. Other distractions and influences due to cognitive factors such as non-associative thoughts about external surroundings, etc. might affect mood states during exercise (Goode and Roth, 1993), however, the intensity of this cognitive task must still match (Fillingim et al., 1989). Yeung (1996), concludes that the effect of exercise on negative emotions is not superior compared to equivalent activities for distraction but in fact is superior when it comes to improving positive mood. If the attitude toward exercise is positive, distraction might reinforce that. Cognitive processes might attenuate negative signals from the body and as a result the overall sensation is primarily positive (this mechanism is discussed further in category 4, PPE).

Another approach might be the theory of sensation seeking, a concept which describes the need for various, novel and complex sensations and the acceptance of accompanied risks (Zuckerman, 1979). Although the four scales (*thrill and adventure seeking, experience seeking, disinhibition, and boredom susceptibility*) suit NE on first impression, the differences found among the three independent factors did not match with recent evidence. While sensation seeking is stronger amongst younger people and men (Zuckerman et al., 1978; Steinberg et al., 2008; Cross et al., 2013), the participants reported the opposite in terms of age, sex, and readiness to assume risk.

Since sensory deprivation reinforces inter alia the urge to move and extrinsic sensory stimuli (Solomon et al., 1957), people might desire similar needs without such need for extremes.

The seeming paradox of this category is that new experiences and impressions, curiosity and excitement are completely opposite to relaxation and cognitive balance or rest which are also comprised in NE. However, both dimensions share in common a positive valence and the contrast to the daily life routine even though they are opposites. The first form of NE describes the positive state of ‘feelings’ in such a situation- high arousal and activation of a person. Seeking sensation, adventure, challenge, or just the desire not only to move but also to gain other experiences in comparison to everyday life might be one explanation for such an emotional response and resulting behavior. The second appearance of NE describes the lower but fair arousal and activation of a person seeking some relaxation and rest. In this particular situation people also described positive emotions in contrast to a stressful daily routine, which meant switching off and letting their thoughts go. In both forms of NE the outcome is nearly the same: positive emotions, enjoyment, and satisfaction. However, the trigger is a different one. Therefore none of the participants described a relaxation effect following a state of excitement or curiosity accompanied by rest.

Positive emotions were intended to promote curiosity and novelty to explore the surroundings for resources (Ekkekakis et al., 2005; Cohn and Fredrickson, 2006) rather than responding to a stimulus demonstrating a strong affinity between NE and positive emotions. Phenomena such as the ‘runners high’ underline the interplay between NE and PPE.

### Perceived Physical Exertion

In relation to exercise-intensity, the emotional response of individuals differs in terms of magnitude, direction, or accountable sources (Ekkekakis and Petruzzello, 1999; Ekkekakis et al., 2000, 2005; van Landuyt et al., 2000; Hall et al., 2002; Ekkekakis, 2003). It is reasonable to assume, that individuals always seek the best emotional outcome and choose the appropriate intensity accordingly, whereas a deviation might lead to less pleasure and enjoyment (Ekkekakis, 2003; Ekkekakis et al., 2005; Ekkekakis and Lind, 2006; Vazou-Ekkekakis and Ekkekakis, 2009). However, some people experience positive emotions at lower levels of intensity and duration of exercise (Ekkekakis and Petruzzello, 1999; Ekkekakis et al., 2000; Reed and Ones, 2006), while some feel comfortable with moderate to high exercise intensity, others will not (Yeung, 1996; van Landuyt et al., 2000; Ekkekakis, 2003). As intensity approaches functional limits, divergent sensations vanished as well as positive valence (Ekkekakis and Petruzzello, 1999; Hall et al., 2002; Ekkekakis, 2003, 2009; Ekkekakis et al., 2005, 2011). Despite these negative emotions some participants (especially those with a higher fitness level) chose demanding intensities and pushed themselves to their limits possibly to generate a higher physical contrast to that of the daily life demand on the body via exhaustion or pain [“hurt so good”(Ekkekakis, 2003)]. Perhaps other influences attenuate the unpleasant sensations associated with strenuous exercise and generate the desire to exercise. Although the main impact on emotions in this domain is physical and increases proportionally with exercise intensity whilst cognitive effects diminish (Ekkekakis, 2003, 2009). However, it still has a cognitive component (e.g., conscience, pride, guilt, etc.; connection to the first category PC).

Another explanation, why people choose intensities that have minimal positive emotions might be due to a *follow-up* or *rebound effect* after the physical activity is finished. In general, exercise is consistently ensued by positive emotions (Tuson and Sinyor, 1993; Yeung, 1996; Ekkekakis, 2003), even if it is at a demanding intensity (Hall et al., 2002). This is despite recent emotional bias during exercise (Ekkekakis and Petruzzello, 1999) which might exceed pre-exercise states independently of intensity (Ekkekakis et al., 2008). This *affective contrast* (Solomon, 1980, 1991) might be due to an interference of two affective dynamic processes with different patterns in terms of effect direction, emergence, and duration. In contrast to each other, the a-process has a negative valence, which is stimulated beyond a certain threshold, then matches the intensity and disappears along with it. Whereas the b-process has a positive valence, is triggered by the first, has a completely opposite bias and appears to be more consistent with slower emergence and decline (Solomon, 1980, 1991). The sensation during exercise is the result of subtraction of both whereas in the recovery-phase the b-process solely determines the emotional outcome. This leads to the conclusion that during high intensity exercise individuals might experience negative emotions, due to a high salient influence of the a-process, but afterward the emotional response is always positive because only the b-process remains. The concept of flow (Csikszentmihalyi, 1975) or phenomena such as the ‘runners high’ might be reflected through the b-process during exercise and mark the threshold where positive emotions occur. However, the a-process might include interoceptive signals which rise as the intensity increases whereas the b-process is charged with cognitive signals which diminish with growing intensity in favor of the first process, which is similar to the dual mode concept (Ekkekakis, 2003, 2009). Another explanation for positive emotions due to the influence of the b-process might be a feeling of relief and alleviation accompanied by biochemical processes inducing regeneration (Hatfield and Landers, 1986). Due to this relationship, positive emotions vanish whilst exercise intensity increases and will return immediately upon exercise cessation explaining the ‘feel-better-effect’ commonly mentioned by participants and people in general (Morgan, 1985). In other words, some individuals may not initially enjoy the activity itself but seek the positive emotions afterward. Statements among those interviewed supported these notions. However, in their review, Rhodes and Kates (2015) did not find any evidence, that post-exercise emotional experience influenced future exercise behavior. Either a lack of sensitivity and power of the method, different mechanics between emotional experience during and after exercise, aforementioned relationships with other facilitators or habitual issues such as the overall exercise experience which might attenuate current emotional influences cause this contrariness which must be clarified through future research.

Relating to the strong difference in terms of sex, references to a higher intensity and especially to exceeding physical limits and pushing oneself hard were noticeably common amongst men. According to the opponent-process model (Solomon, 1980, 1991), men might have a higher resistance to the a-process or potent factors like PC (category 1) boosting the b-process, whilst women do not. Reinforced by a weaker fitness level, they perceive exercise as more strenuous and less pleasant and may avoid higher intensity exercise (Ekkekakis and Petruzzello, 1999; Ekkekakis and Lind, 2006).

The difference in terms of sport type is small. While participants in individual recreational sports could decide autonomously, those people in a team or group must adapt to the collective intensity expected–this normally results in a decrease of positive valence (Dishman et al., 1994; Ekkekakis and Petruzzello, 1999; Vazou-Ekkekakis and Ekkekakis, 2009) as well as it being due to supra-threshold intensities (Ekkekakis et al., 2011). However, the slightly higher frequency in positive emotion-related statements of team-sports participants suggests that team-dynamics as well as providing a distraction attenuate the interoceptive signals that improve the resistance to the a-process, raise the threshold and reinforce the b-process during exercise and later on as well.

The age groups show a strong decline in positive emotions related to exercise intensity. A transition from interoceptive dominance at a younger age to cognitive dominance can be expected. According to the opponent-process model (R. L. Solomon, 1980, 1991), a higher resistance to the a-process or a higher sensitivity to the b-process and lower self-awareness at a younger age might reflect the urge to move and the need for vigorous exercise. An inverted relationship at an older age with higher maturity induces avoidance to strenuous exercise not only because of health issues, restrictions, and ailments but actually increased rationalizing results in the motivation to do exercise for other reasons. As one ages the threshold for desired high intensity exercise seems to weaken which is reinforced by a decreasing fitness level influencing the overall exercise experience (Ekkekakis et al., 2005).

### Self Determination Theory

According to the self determination theory (Deci and Ryan, 2010) humans have three psychological needs which are essential for intrinsic motivation to adopt and adhere behavior (Kwasnicka et al., 2016) or an activity in particular: the need for competence, relatedness, and autonomy. Although the theory is focused on intrinsic and extrinsic motivation and this study is focused on facilitators of positive emotions, the similarities are striking. The perception of competence and affiliation plays an important role to elicit positive emotions through satisfaction of these basic needs. This leads to the assumption, that positive emotions and intrinsic motivation are related constructs. Furthermore it is not surprising that the need for autonomy was not as strong as the other two among the interviews since all participants were voluntary involved in recreational sports in their leisure time and autonomy was naturally present. Nevertheless there are some characteristics of autonomy in common with NE, such as self-control or the ability to choose, e.g., skiing or sailing a self-chosen course or running at one’s own desired pace. Additionally NE is described as contrast to the repetition of daily routine which contains commitments and limited autonomy. However, when this is restricted due to a forced interruption (e.g., such as injuries or appointments) this negate any positive emotions. The selective coding process revealed that even a high level of perception of competence or relatedness seemed to both facilitate positive emotions even more if this perception was further increased, e.g., one is performing well and later on is victorious or teammates become close friends. In contrast references to autonomy were more common when it was threatened which indicates, that in this case it is a much stronger facilitator for negative emotions. However, as an avenue for future work the different function of negative emotions (e.g., survival, avoid harm) and positive emotions (e.g., adaptation, extend resources) require separate attention since negative emotions influence behavior in a different way (Cohn and Fredrickson, 2006).

### Implications

This study is a novel and perhaps promising approach to illuminate facilitators of positive emotions to explain exercise maintenance. Firstly, these findings encourage continuation of practical application studies to advance the knowledge about potential facilitators of emotional responses to exercise. Secondly, based on these results, interventions could be developed which enhance the emotional response during exercise by manipulating these four facilitators (e.g., due to a corresponding design or instructor behavior). Thirdly, recommendations for the design and evaluation of exercise programs can be made in so far as to reduce the drop-out rate and to increase commitment and adherence to sport. In an experimental study, Jekauc (2015) could show that emotions could be manipulated to increase adherence in exercise. However, further quantitative studies are needed to explicitly test the relevance of the factors discovered here.

### Strength and Limitations

This study has some strengths and limitations. For the best approach to the emotional dimension, Dishman (1994) and Rhodes et al. (2009) proposed a qualitative and therefore inductive design because qualitative methods meant having a higher capability to illuminate the different sources of enjoyment by including a broad spectrum of influences as expected in the sporting environment (Scanlan and Simons, 1992). Also the value of information is much higher if participants are able to express their feelings with their own words. They might reveal factors which might otherwise be missed. The semi structure interview guide guaranteed a uniform approach. For a qualitative study, it has quite a large sample of 24 participants which is heterogeneous as sex, age, and type of sport are systematically varied. Furthermore the analysis via Grounded Theory ensured systematical categorization.

However, the sample is not large enough to be a representative sample. Despite a progressive but sometimes deficient differentiation of constructs and convergence of several theories, terms in the literature are still used inconsistently which hinders the ability to compare. Presented findings were drawn from recreational sport and exercise and might not count for specific aspects such as competitive sport or for a broader remit such as physical activity.

## Conclusion

In this study, four facilitators of positive emotions during exercise and recreational sport were identified: *PC*, *PSI*, *NE*, and *PPE*. These results could provide the starting point for the development of interventions aiming at the promotion of positive emotional states in sport in order to increase maintenance and adherence to sport and exercise. Future steps will be to design intervention studies according to the findings of this study and to test their effectiveness in experimental studies.

## Author Contributions

BW is the main author of the submitted article and responsible for the overall conception and design of this manuscript. He is also responsible for the data collection, analysis, and interpretation. DJ is the second author and contributed to the design of the study, he was involved in the interpretation and provided edits to the paper. Both authors read and approved the final manuscript.

## Conflict of Interest Statement

The authors declare that the research was conducted in the absence of any commercial or financial relationships that could be construed as a potential conflict of interest.
